# Cardiac autonomic neuropathy in type 1 and type 2 diabetes patients

**DOI:** 10.1186/s12883-018-1125-1

**Published:** 2018-08-27

**Authors:** Anca Moţăţăianu, Smaranda Maier, Zoltan Bajko, Septimiu Voidazan, Rodica Bălaşa, Adina Stoian

**Affiliations:** 1 Department of Neurology, University of Medicine and Pharmacy Târgu Mureş, Gh Marinescu 50, 540136 Târgu Mureş, Romania; 2 Department of Epidemiology, University of Medicine and Pharmacy Târgu Mureş, Târgu Mureş, Romania; 3 Department of Pathophysiology, University of Medicine and Pharmacy Târgu Mureş, Târgu Mureş, Romania

**Keywords:** Cardiac autonomic neuropathy, Type 1 diabetes, Type 2 diabetes, Microvascular complication, Macrovascular complication, Cardiovascular risk factors, Glycaemic control

## Abstract

**Background:**

Cardiac autonomic neuropathy (CAN) in diabetes is among the strongest risk markers for future global and cardiovascular mortality**.** The aim of this study was to analyse CAN prevalence and to compare the associations between CAN, the glycaemic control, cardiovascular risk factors, peripheral neuropathy, retinopathy and macroangiopathy in patients with type 1 (T1DM) and type 2 diabetes mellitus (T2DM).

**Methods:**

One hundred ninety-five diabetic patients were included in this study. All patients were evaluated for detection of CAN (with standardised cardiovascular reflex tests), diabetes-related microvascular complications (polyneuropathy, retinopathy), common carotid artery intima-media thickness (IMT) and ankle-brachial index (ABI).

**Results:**

The prevalence of CAN was 39.1% in T2DM and 61.8% in T1DM patients. Multivariate logistic regression analysis demonstrated that in T2DM, the odds [OR (95% confidence intervals)] of CAN increased with diabetes duration [1.67(1.42–1.92)], HbA1c [1.74(1.34–2.27)], cholesterol [1.01(1.00–1.01)], triglycerides [1.01(0.99–1.00)], smoking [2.35(1.23–4.49)], systolic blood pressure [1.01(1.00–1.03)], BMI [1.16(1.08–1.24)], glomerular filtration rate [0.91(0.88–0.93)], peripheral neuropathy [25.94(11.04–44.25)], retinopathy [13.13(3.03–84.73)] and IMT [10.12 (7.21–15.32)]. In T1DM, the odds of CAN increased with diabetes duration [1.62(1.13–2.31)], HbA1c [4.49(1.27–15.9)], age of patients [1.14(1.03–1.27)], glomerular filtration rate [0.94(0.89–0.99)], peripheral neuropathy [31.6(4.5–45.8)] and IMT [5.5(2.3–8.3)].

**Conclusion:**

This study indicated that CAN is a more frequent complication in T1DM. Apart from glycaemic control, the existence of CAN is associated with potentially modifiable cardiovascular risk only in T2DM patients. The presence of other micro- and macrovascular complications increases the probability of having CAN in both types of DM (but more pronounced in T2DM).

## Background

Diabetes is a problem of major concern and has been characterised as the primary health care challenge of the twenty-first century. The prevalence of diabetes, along with its complications, has risen rapidly. Currently, type 2 diabetes mellitus (DM) is an epidemic development throughout the world. Data from the International Diabetes Federation (IDF) show that in 2015 almost 5 million patients across the world died due to diabetes and its complications. The prevalence of type 1 diabetes mellitus (T1DM) shows a rapid increase as well as the rise of type 2 diabetes mellitus (T2DM) in younger patients [1].

Cardiac autonomic neuropathy (CAN) is one of the most overlooked of all serious complications of diabetes. Although silent in the earlier stages, it is a powerful predictor of mortality risk in diabetic patients and is a major challenge for all physicians dealing with people suffering from diabetes. Patients with CAN have a five-fold increased risk of mortality due to a high-risk of cardiac arrhythmias, silent myocardial ischaemia and sudden death. The burden of DM is reflected not only in the increasing number of patients but also in the growing number of premature deaths due to diabetes [2–4].

The purpose of this study was to evaluate the CAN characteristics in T1DM versus T2DM patients and to identify the relationship between CAN, cardiovascular risk factors (CVRF) and other microvascular and macrovascular complications. Currently, more studies are required when considering the global increase of DM in order to improve the strategies for fresh CAN prevention.

## Methods

This was a cross-sectional study in which 212 consecutive inpatients with T1DM and T2DM were selected among diabetic subjects who presented to the Diabetes Department of the TirguMures University Hospital (Romania)*.* Exclusion criteria were as follows: presence of cardiac arrhythmia, heart blockage, clinical coronary artery disease, presence of thyroid disease, presence of hypo- or hyperglycaemia in the previous 24 h, presence of acute illness, severe systemic disease, medication that affects the autonomic nervous system (anti-arrhythmic medication, antidepressants, antihistamine and sympathomimetic cough preparations), alcohol abuse, use of neurotoxic medication or malignant disease, history of diabetic ketoacidosis and other secondary causes of diabetes [3]. Based upon these exclusion criteria, 17 patients were excluded from the study group. The majority of our patients were hospitalised for periodical control, not for acute comorbidities. This study protocol was approved by the University of Medicine and Pharmacy Tirgu Mures Review Board, and all subjects gave their written informed consent.

Clinical examination was performed and stress on heart rate, systolic and diastolic blood pressure (SBP/DBP), body weight, waist circumference and body mass index (BMI) were recorded*.* Complete blood count and chemistry tests (including complete renal, hepatic and other metabolic testing panels) were collected in the morning after an overnight fast. Glycated haemoglobin (HbA1c) was determined by high-performance liquid chromatography with a non-diabetic reference range of 4.1–6.0. Renal function was assessed by estimated glomerular filtration rate (eGFR), which was calculated using the Modification of Diet in Renal Disease study (MDRD) formula [5].

Diabetic retinopathy was evaluated by an experienced independent ophthalmologist. Direct funduscopy was performed on dilated pupils, and the findings were classified as normal, pre-proliferative retinopathy (including maculopathy) or proliferative retinopathy. For statistical analysis, we considered those patients without retinopathy with normal funduscopy and pre-proliferative retinopathy, and patients with retinopathy with proliferative retinopathy.

Neuropathic symptoms were assessed based upon neuropathy symptom scores as previously described [6].

Peripheral nerve function was assessed using nerve conduction studies (NCS), by an electrodiagnostic protocol as recommended by the American Diabetes Association [7]. For each patient, NCS were performed bilaterally on the median, ulnar, peroneal, tibial and sural nerves according to standard techniques [8]. NCSs were performed with a four-channel electromyography (EMG) apparatus (Neuro-MEP-4, Russia) with surface electrodes. Reduced amplitudes in the motor or sensory responses less than the normal limit (mean–2 standard deviations, SD) and slowness in the motor or sensory conduction velocity less than the normal limit (mean–2 SD) were identified as abnormal values [8]. When two or more nerves were abnormal, NCS were considered abnormal according to the Mayo Clinic staging criteria [9]. The patients were classified as having subclinical peripheral neuropathy in the absence of signs or symptoms of neuropathy if they had abnormal NCS. They were classified as having confirmed peripheral neuropathy in the presence of abnormal NCS and signs or symptoms of polyneuropathy (PNP), and they were considered to have no PNP if NCS were normal with no symptoms or signs of neuropathy on clinical examination [10].

Patients were requested to avoid strenuous physical exercise in the 24 h preceding the cardiovascular testing and to avoid smoking, eating or coffee consumption for at least 2 h prior to autonomic testing. All antidiabetic and other medications were administered at the end of the examination.

Cardiovascular autonomic reflex tests were performed by one examiner early in the morning according to Ewing’s method, which includes a battery of five noninvasive autonomic tests [11]. Testing of autonomic parasympathetic dysfunctions was assessed by the heart rate variability (HRV) to slow deep breathing with a rate of six breaths per minute Valsalva manoeuvring and a postural change from lying to standing. HRV was assessed from electrocardiographic recordings of R-R intervals automatically using an ELI 350 electrocardiograph system (Mortara Instrument Inc., Milwaukee, USA). For HRV to the Valsalva manoeuvring, the ratio between the longest R-R interval to the shortest R-R interval was assessed during forced exhalation into a mouthpiece of a manometer to 40 mmHg for 15 s. The HRV to postural changes was evaluated by the ratio of the longest R-R interval during beats 20–40 after standing to the shortest R-R interval during beats 5–25 after standing and HRV to deep breathing was asseseed by recording the difference between the maximum and minimum heart rates (beats/minute). These tests were performed using technique-specific normative data as previously described [12]. The test results of the deep-breathing test were interpreted according to normal age-related values [13]. Sympathetic dysfunction was assessed by measuring blood pressure response to postural change from lying to standing and to sustained handgrip. Orthostatic hypotension was defined as a reduction of systolic blood pressure of at least 20 mmHg, or diastolic blood pressure of at least 10 mmHg, within 3 min of standing. The blood pressure measurement point while standingwas at heart level. Details of these assessments of cardiovascular autonomic function have been previously described [3]. CAN was defined as the presence of at least two abnormal standard tests [10]. The patients were divided into two groups according to the presence or absence of CAN.

The common carotid artery intima-media thickness (IMT) was assessed using ultrasonography (Siemens Accuson Antares Ultrasound System) on both bilateral common carotid arteries with a linear array 5-mHz transducer as reported previously [14]. Scanning was performed at three different longitudinal projection sites (anterior-oblique, lateral and posterior-oblique). The IMT was measured at the thickest portion of the scanning area, and at two other points: 1 cm upstream and 1 cm downstream from the site of greatest thickness. The mean of these three IMT measurements was used as the individual’s IMT. We also evaluated the ankle-brachial index (ABI) with a handheld 5-mHz Doppler device (HI Dop Vascular Doppler set) in all patients.

## Statistical analyses

Statistical analyses were performed using MedCalc Software (Version 12.3.0 bvba, Mariakerke, Belgium). Data were considered as nominal or quantitative variables. Nominal variables were characterized using frequencies. Quantitative variables were tested for normality of distribution using Kolmogorov-Smirnov test and were characterized by median and range or by mean and standard deviation (SD), when appropriate. A chi-square test was used in order to compare the frequencies of nominal variables. Quantitative variables were compared using t test, Mann-Whitney test, when appropriate. Multivariate analysis was carried out using linear regressions. We used as dependent variable the CAN: CAN+ code 1 vs CAN- code 0. We used the Bonferroni correction in order to account for multiple comparisons. The level of statistical significance was set at *p* < 0.05.

## Results

### General characteristics of study patients

Baseline characteristics of the studied patients are shown in Table 1. At the time of the study, the patients with T2DM were older than those with T1DM and had a shorter duration of diabetes, but had better glycaemic control than the T1DM patients group as reflected by HbA1c levels. Despite the better glycaemic control in T2DM, the prevalence of hypertension, BMI, triglyceride levels and abdominal obesity reflected by waist-to-hip ratios were significantly higher in T2DM patients. The incipient macrovascular complications reflected by IMT were more evident in T2DM patients than in those with T1DM, but the prevalence of the advanced microvascular complications, clinical polyneuropathy and proliferative retinopathy were significantly higher in T1DM patients.

**Table 1:** Baseline characteristics of studied patients

Variable	DM type 1	DM type 2	*p* value
Patients number	34	161	
Male/Female, nr (%)	12 (35.3)/22 (64.7)	76 (47.2)/85 (52.8)	0.20^#^
Age (years)	36.7 ± 9.5	58.1 ± 8.2	0.0001**
Age at diabetes diagnosis (years)	22.2 ± 7.5	49.8 ± 9.2	0.0001**
Diabetes duration (years)	14.5 (1–27)	6 (1–37)	0.0001*
Diabetes duration			
< 5 years, nr (%)	4 (11.8)	61 (37.9)	0.01^#^
5–10 years, nr (%)	6 (17.6)	28 (17.4)	0.78^#^
11–15 years, nr (%)	10 (29.43)	54 (33.5)	0.54^#^
> 15 yers, nr (%)	14 (41.2)	18 (11.2)	0.001^#^
Body mass index (kg/m2)	22.6 ± 3.9	30.8 ± 5.3	0.001**
Systolic BP(mmHg)	123.6 ± 13.8	143.4 ± 19.6	0.001**
Diastolic BP (mmHg)	72.6 ± 10.4	81.4 ± 9.9	0.001**
Pulse pressure	51.8 ± 10.4	61.5 ± 16.1	0.001**
Hypertension (yes), nr (%)	9 (26.5)	106 (65.8)	0.001^#^
Ex-smokers, nr (%)	21 (61.8)	92 (57.1)	0.32^#^
Smokers (yes), nr (%)	13 (38.2)	69 (42.9)	0.42^#^
< 20 cigarettes/day, nr (%)	10 (71.4)	33(47.8)	0.38^#^
> 20 cigarettes/day, nr (%)	4 (28.6)	36 (52.2)	0.38^#^
Triglycerides (mg%)	142.0 (76–478)	178 (60–1100)	0.003*
HgbA1c	9.2 ± 1.3	8.3 ± 1.4	0.004**
FPG (mg%)	231.8 ± 63.5	183.9 ± 64.0	0.001**
eGFR (ml/min per 1.73 m2)	71.1 ± 24.7	75.6 ± 20.5	0.26**
PNP			
Clinical, nr (%)	22 (64.7)	68 (42.2)	0.02^#^
Subclinical, nr (%)	0 (0.0)	46 (28.6)	0.005^#^
No PNP, nr (%)	12 (35.3)	44 (29.2)	0.78^#^
Retinopathy			
Proliferative, nr (%)	8 (23.5)	10 (6.2)	0.002^#^
Preproliferative, nr (%)	12 (35.3)	49 (30.4)	0.82^#^
No retinopathy, nr (%)	14 (41.2)	102 (63.4)	0.001^#^
ABI*	1 (0.75–1.3)	0.95 (0.75–1.35)	0.32^#^
QTc	431.5 ± 37.2	426.4 ± 31.6	0.41**
IMT	0.76 ± 0.19	0.91 ± 0.19	0.001**
Waist to hip ratio	0.81 ± 0.02	0.90 ± 0.08	0.001**
Abdominal circumference	83.8 ± 9.9	104.6 ± 12.3	0.001**

Data were expressed as mean ± SD, − student t test**; median (range) - Mann Whitney test* and no (%) - chi square test^**#**^

*CAN*- cardiac autonomic neuropathy, *BP*- blood pressure, *FPG* - fasting plasma glucose, HgbA1c-glycosylated hemoglobine, *eGFR* - estimated glomerular filtration rate, *PNP*-peripheral neuropathy, *ABI*- ankle-brachial index, *IMT* - intima-media thickness, *QTc*- corrected QT interval

### Comparing the study patients with and without CAN

Table 2 compares the diabetic patients with and without CAN between the T1DM and T2DM.

**Table 2:** Characteristics of the study patients according to the presence (CAN+) or absence of CAN (CAN−)

Variable	DM type 1			DM type 2		
	CAN -	CAN +	*p* value	CAN -	CAN +	*p* value
Nr (%)	13 (38.2)	21(61.8)		98 (60.9)	63 (39.1)	
Male/Female, nr (%)	5(38.5)/8 (61.5)	7 (33.3)/14 (66.7)	0.76^**#**^	47 (48.0)/51 (52.0)	29 (46.0)/34 (54.0)	0.81^**#**^
Age (years)	30.8 ± 8.2	40.3 ± 8.5	0.003**	57.4 ± 8.5	59.1 ± 7.7	0.22**
Age at diabetes diagnosis (years)	24.4 ± 7.3	20.7 ± 7.4	0.16**	53.1 ± 8.6	45.0 ± 7.9	0.0001**
DM duration (years)	7 (1–11)	22 (6–27)	0.0001*	4 (1–15)	12 (5–37)	0.0001*
DM duration						
< 5 years, nr (%)	4 (30.8)	0 (0.0)	0.02^**#**^	61 (62.2)	0 (0.0)	0.0001^**#**^
5–10 years nr (%)	8 (61.5)	2 (9.5)	0.04^**#**^	8 (8.2)	20 (31.7)	0.002^**#**^
11–15 years, nr (%)	1 (7.7)	5 (23.8)	0.52^**#**^	29 (29.6)	25 (39.7)	0.24^**#**^
> 15 years, nr (%)	0 (0.0)	14 (66.7)	0.001^**#**^	0 (0.0)	18 (28.6)	0.0001^**#**^
BMI (kg/m2)	21.1 ± 4.1	23.5 ± 3.5	0.08**	29.3 ± 4.9	33.2 ± 5.1	0.0001**
SBP (mmHg)	119.4 ± 11.3	126.3 ± 14.8	0.16**	140.6 ± 18.4	149.9 ± 20.7	0.01**
DBP (mmHg)	70.7 ± 7.2	73.9 ± 11.9	0.38**	80.9 ± 9.4	82.1 ± 10.7	0.44**
Pulse pressure	48.7 ± 8.2	53.8 ± 11.3	0.16**	59.5 ± 15.4	64.6 ± 16.5	0.04**
Hypertension (yes)	1 (7.7)	8 (38.1)	0.051^**#**^	62 (63.3)	44 (69.8)	0.39^**#**^
Nonsmokers	7 (53.8)	14 66.7)	0.45^**#**^	64 (65.3)	28 (44.4)	0.03^**#**^
Smokers	6 (46.2)	7 (33.3)	0.45^**#**^	34 (34.7)	35 (55.6)	0.02^**#**^
< 20cigarettes/day	5 (38.5)	5 (23.8)	0.5^**#**^	17 (17.3)	16 (25.4)	0.78^**#**^
> 20cigarettes/day	2 (15.4)	2 (9.5)	0.5^**#**^	17 (17.3)	19 (30.2)	0.54^**#**^
Triglycerides (mg%)	142 (76–188)	143 (78–478)	0.22*	154.5 (60–996.0)	204 (95–1100)	0.001*
Cholesterol (mg%)	170.5 ± 26.3	200.6 ± 68.3	0.14**	204.5 ± 29.1	228.7 ± 49.1	0.002**
HgbA1c	8.4 ± 0.72	9.6 ± 1.37	0.01**	7.9 ± 1.4	9.1 ± 1.2	0.001**
FPG (mg%)	229.2 ± 55.8	233.4 ± 69.2	0.85**	173.6 ± 68.2	199.7 ± 53.7	0.01**
eRFG	86.9 ± 17.4	60.7 ± 23.6	0.001**	85.6 ± 17.2	60.4 ± 14.6	0.0001**
PNP						
Clinical	3 (23.1)	19 (90.5)	0.0001^**#**^	16 (17.5)	52 (82.5)	0.0001^**#**^
Subclinical	0 (0.0)	0 (0.0)	–	40 (40.8)	6 (9.5)	0.0001^**#**^
No PNP	10 (76.9)	2 (9.5)	0.0001^**#**^	42 (42.9)	5 (7.9)	0.0001^**#**^
Retinopathy						
Proliferative	0 (0.0)	8 (38.1)	0.003^**#**^	2 (2.0)	8 (12.7)	0.002^**#**^
Preproliferative	0 (0.0)	12 (57.2)	0.001^**#**^	12 (12.2)	37 (58.7)	0.0001^**#**^
No rethinopathy	13 (100.0)	1 (4.8)	0.001^**#**^	84 (85.7)	18 (28.6)	0.0001^**#**^
ABI	1.02 (0.91–1.1)	0.92 (0.75–1.3)	0.30*	1.01 (0.78–0.14)	0.86(0.75–1.35)	0.0001*
QTc	402.8 ± 33.4	449.2 ± 27.4	0.001**	412.7 ± 27.6	447.8 ± 25.1	0.0001**
IMT	0.60 ± 0.13	0.86 ± 0.16	0.001**	0.82 ± 0.17	1.03 ± 0.15	0.0001**

Data were expressed as mean ± SD, − student t test**; median (range) - Mann Whitney test* and no (%) - chi square test^**#**^

*CAN* - cardiac autonomic neuropathy, *BP*- blood pressure, *FPG* - fasting plasma glucose, HgbA1c-glycosylated hemoglobine, *eGFR* - estimated glomerular filtration rate, *PNP*- peripheral neuropathy, *ABI*- ankle-brachial index, *IMT* - intima-media thickness, *QTc*- corrected QT interval

Of 34 patients with T1 DM, 21 (61.8%) were diagnosed with CAN. T1DM patients with CAN had a higher duration of diabetes, poorer glycaemic control as indexed by HbA1c levels, a lowere GFR, a higher prevalence of clinical polyneuropathy and proliferative retinopathy and an increased IMT than patients without CAN.

Of 161 patients with T2DM, 63 (39.1%) were diagnosed with CAN. Compared with T2DM patients without CAN, the T2DM patients with CAN were younger at the time of diabetes diagnosis and had a longer duration of diabetes, poorer glycaemic control reflected by HbA1c levels, a significantly higher BMI, a significantly higher systolic blood pressure level, higher cholesterol levels and were more frequently smokers. The prevalence of pre-proliferative retinopathy, proliferative retinopathy and clinical polyneuropathy were significantly higher in the group of T2DM patients with CAN than in those without CAN.

### Logistic regression analysis

Univariate logistic regression analysis (Table 3) was performed to identify determinants of CAN in both types of DM. In T2DM patients, the odds [OR (95% confidence intervals)] of CAN increased with age at diabetes diagnosis, diabetes duration, BMI, smoking, systolic blood pressure, cholesterol level, triglyceride level, HbA1c level, increased IMT, existence of PNP and retinopathy.

**Table 3:** Univariate linear regression analysis: ORs and 95% CIs for CAN in T1 DM and T2 DM patients

CAN in T1DM patients			CAN in T2DM patients	
Variable	OR (CI 95%)	*p*-value	OR (CI 95%)	*p*-value
Male/Female, nr (%)	0.80 (0.18–3.37)	0.76	0.92 (0.49–1.75)	0.81
Age (years)	1.14 (1.03–1.27)	0.0009	1.02 (0.98–1.06)	0.22
Age at DM diagnosis (years)	0.93 (0.83–1.03)	0.17	0.89 (0.85–0.94)	< 0.0001
DM duration (years)*	1.62 (1.13–2.31)	0.0007	1.67 (1.42–1.92)	0.0001
BMI (kg/m2)	1.19 (0.96–1.48)	0.09	1.16 (1.08–1.24)	0.0003
SBP (mmHg)	1.04 (0.98–1.10)	0.16	1.01 (1.00–1.03)	0.02
DBP(mmHg)	1.01 (0.96–1.10)	0.38	1.004 (0.97–1.03)	0.44
Hypertension (yes)	7.38 (0.80–68.1)	0.07	1.34 (0.68–2.68)	0.39
Smoking (yes vs. no)	0.62 (0.15–2.61)	0.52	2.35 (1.23–4.49)	0.0009
Cholesterol (mg%)	1.01 (0.98–1.04)	0.14	1.01 (1.00–1.01)	0.004
Triglyceride (mg%)	1.01 (0.99–1.02)	0.16	1.00 (0.99–1.00)	0.03
HgbA1c	4.49 (1.27–15.9)	0.01	1.74 (1.34–2.27)	< 0.0001
FPG (mg%)	1.01 (0.99–1.01)	0.85	1.00 (1.00–1.01)	0.01
eRFG (ml/min/1.73 m2)	0.94 (0.89–0.99)	0.007	0.91 (0.88–0.93)	< 0.0001
ABI	1.13 (0.01–76.01)	0.95	0.01 (0.00–0.20)	0.001
QTc	1.05 (1.01–1.1)	0.007	1.20 (1.03–1.07)	< 0.0001
IMT	5.5 (2.3–8.3)	0.007	10.12 (7.21–15.32)	< 0.0001
RD (yes vs no)	1.25 (0.56–1.9)	0.88	13.13 (3.03–84.73)	0.002
PNP (yes vs no)	31.6 (4.5–45.8)	0.001	25.94 (11.04–44.25)	0.0001

*BP*- blood pressure, *FPG* - fasting plasma glucose, HgbA1c-glycosylated hemoglobine, *eGFR* - estimated glomerular filtration rate, *PNP*- peripheral neuropathy, *ABI*- ankle-brachial index, *IMT* - intima-media thickness, *QTc*- corrected QT interval, *- median range

For T1DM patients, the significant predictors for the existence of CAN were age of patients, diabetes duration, HbA1c, peripheral neuropathy and IMT. The traditional CVRF (hypertension, obesity, smoking, dyslipidemia) had no effect on the risk of developing CAN in T1DM patients.

## Discussion

The differences between T1DM and T2DM in terms of prevalence, disease mechanism (deficiency of insulin versus insulin resistance), age of onset, typical conformation of the patient and the treatment are well known. Five percent of patients with DM have T1DM, a disease mostly seen in children and young adults, which is characterised by autoimmune destruction of beta cells with loss of insulin production. The remaining 95% patients have T2DM, a metabolic disease with high pancreatic insulin production in the setting of insulin resistance. In a recent meta-analysis, the authors concluded that the neuropathy in T1DM and T2DM are substantially different complications with disparate mechanisms. Glucose control in T1DM has a large effect on prevention of neuropathy, but in T2DM, glucose control has a small effect on the prevention of neuropathy [15].

In a recent study that evaluated long-term clinical outcomes and survival in young-onset T1DM compared with T2DM at the same age at onset, the results established that the young-onset T2DM was the most lethal phenotype of diabetes because is associated with a greater mortality, more diabetic complications and unfavourable cardiovascular disease risk factors when compared to T1DM [16].

In T1DM, there is clear evidence for genetic predisposition but also strong evidence for an autoimmune mechanism for destruction of the beta cells leading to absolute dependence on insulin treatment. Neurons and pancreatic beta cells have a neuroectodermal origin and therefore share common antigens, especially in the early stage of evolution. Granberg et al. [17] provided epidemiological data that support the implication of autoimmunity in autonomic neuropathy in T1DM by demonstrating the existence of antibodies against the autonomic nervous system (sympathetic ganglion, vagus nerve, adrenal medulla) in T1DM patients with autonomic neuropathy.

In our study, poor glycaemic control and the long duration of diabetes were the key risk factors for developing CAN in either T1DM or T2DM. We found that the patients with T1DM have a longer duration of diabetes compared with T2DM patients (22 vs 12.5 years). This difference can be explained by the fact that the patients with T2DM often have a history of many years without symptoms during which blood glucose peaks occur unnoticed, but diabetes is not yet diagnosed and treated. So, in T2DM, the diabetes complications may exist at the time of initial presentation, but in T1DM, there is likely to be a window between initial diagnosis and the onset of organ damage [18].

Poor glycaemic control is a major risk factor for the development and progression of CAN in both types of DM. In our study, poor glycaemic control was associated with the increased risk of having CAN. This result suggests that glycaemic control is a more important driver of cardiac autonomic dysfunction in T1DM than in T2DM. Brownlee [19] demonstrated that the high blood glucose level in the past determined the risk for later diabetic complication. Due to the asymptomatic period in T2DM with inadequate control of hyperglycaemia before the establishment of the diagnosis, further diabetic complications will occur later despite the optimal glycaemic control. This phenomenon is called ‘hyperglycaemic or metabolic memory’, and it is responsible for the initial damage that occurs very early on, even before diabetes has been initially diagnosed. Because we evaluated glycaemic control by HbA1c levels, which reflects the average blood glucose level over the past 3 months, a possible explanation of the differences in OR between T1DM and T2DM patients could be not that our T2DM patients have better glycaemic control in the past 3 months, but a history of ‘silent’ and untreated hyperglycaemia, which plays a major role in hyperglycemia-induced late complications of T2DM.

The Action to Control Cardiovascular Risk in Diabetes (ACCORD) study on 10,251 participants demonstrated that in the patients with T2DM and high cardiovascular risk or preexisting cardiovascular and microvascular damage, the mortality rate was increased in the arm receiving intensive treatment with forced HbA1c reduction. Also, the ACCORD study demonstrated that neuropathies (somatic and autonomic) are significant risk factors for cardiovascular disease, and this particular group of patients represents a high-risk group in which intensive glucose control should be well-balanced against the mortality risk [20, 21]. The basic approach for living with DM and having as few complications as possible is to start treatment immediately after onset of the diabetes with the purpose of achieving metabolic control as much as possible. The actual trend throughout the world is to restrict the prognostic perspective of diabetes to the HbA1c value, but this is not justified by the complex mechanism implicated in vascular complications of DM.

In T1DM, two important epidemiological studies, Diabetes Control and Complications Trial (DCCT) and Epidemiology of Diabetes Interventions and Complications study (EDIC), demonstrated that early intensive glycaemic control can decrease the incidence of CAN, and this protective effect persisted for more than 14 years after the end of the study despite the disappearance of intensive glycaemic control [22, 23]. In a recent Cochrane meta-analysis, it was demonstrated that enhanced glucose control significantly prevents the development of clinical neuropathy only in T1DM [24].

In T2DM, the effect of glycaemic control was not so evident. The United Kingdom Prospective Diabetes Study (UKPDS) on 3867 recently diagnosed T2DM patients demonstrated that at 10 years from inclusion in the study, there were no differences on developing neuropathy between the group with intensive glycaemic control versusthe group with conventional glycaemic control, irrespective of other CVRF. In other studies that followed the UKPDS study (the VA Cooperative study, Duckworth study and Steno-2 study), intensive glycaemic control resulted in a small decrease in diabetic neuropathy incidence, suggesting that in T2DM, factors that are independent from glycaemic control are responsible for the damage of somatic and autonomic nervous system. In the Steno-2 study, there was clear evidence that intensive pharmacological treatment targeting hypertension, hyperlipidemia and microalbuminuria combined with lifestyle changes (diet, smoking cessation and physical exercise) reduced the risk of autonomic neuropathy over the course of almost 8 years of follow-up [25–28].

In the present study, we found that among T2DM patients, the odds of CAN increased with the existence of traditional CVRF (hypertension, smoking, obesity, higher cholesterol level), but CVRF had no effect on cardiac autonomic dysfunction in T1DM patients. Smoking was associated with increased odds of CAN among T2DM patients in our study, but there was no significant association between smoking and CAN in T1DM patients. Although there are no data to explain the effect of smoking on autonomic function in T2DM patients, the studies performed on the non-diabetic population demonstrated that smoking is associated with autonomic dysfunction related to increased oxidative stress and increased inflammatory activity [29].

Our results confirm that in T2DM when CVRF are associated with poor glycaemic control, the risk of developing CAN increased. In order prevent or to slow the progression of CAN, improving glycaemic control, lifestyle changes and cardiovascular risk factor management are the mainstays of treatment,but in T1DM patients, our results are not in accordance with previous studies that demonstrated the associations between CAN and CVRF [30, 31]. This observed difference between T1DM and T2DM patients can be explained by (a) implication of hyperglycaemia and autoimmune mechanisms in developing CAN in T1DM and of hyperglycaemia and CVRF in developing CAN in T2DM or (b) because of the small number of T1DM patients in our study, which can be accepted as a limitation.

In the group of T1DM and T2DM patients, the risk for developing CAN increased in the presence of peripheral neuropathy, retinopathy and accelerated atherosclerosis (reflected by increased IMT and decreased ABI). These associations between cardiac autonomic dysfunction and micro- and macrovascular complications were more evident in T2DM. Microvascular complications of DM share a common pathogenetic factor with atherosclerosis represented by functional disturbances in the vascular endothelium induced by hyperglycaemia and increased oxidative stress. Endothelial dysfunction is considered to be an early stage and precursor of atherosclerosis. Our results are consistent with previous data from Yokoyama and coworkers [32], who reported a positive relationship between diabetic neuropathy (including autonomic neuropathy), increased IMT and arterial stiffness assessed by brachial-ankle pulse wave velocity in T2DM patients. In previous DDCT and EDIC studies on T1DM patients, it was demonstrated that microvascular complication per se conferred an independent risk for macrovascular disease [33, 34].

## Conclusions

As the incidence of diabetes rises, so too does the requirement for healthcare, and in order to prevent CAN in patients with T1DM, we must focus on glycaemic control, but in T2DM we should focus not only on glycaemic control but also on improving adherence to cardiovascular risk factor intervention. In T2DM patients, enhanced glycaemic control can delay development of CAN but increase the risk of severe hypoglycaemic episodes, which need to be taken into account when evaluating the risk/benefits ratio. There is a need for further studies to discover the optimal level of glycaemic control in order to reduce the development of CAN without increasing the risk of death.

## Abbreviations

ABI: ankle-brachial index
ACCORD: The Action to Control Cardiovascular Risk in Diabetes
BMI: body mass index
CAN: cardiac autonomic neuropathy
CVRF: cardiovascular risk factors
DBP: diastolic blood pressure
DCCT: Diabetes Control and Complications Trial
EDIC: Epidemiology of Diabetes Interventions and Complications
eGFR: estimated glomerular filtration rate
EMG: electromyography
FPG: fasting plasma glucose
HbA1c: glycated haemoglobin
HRV: heart rate variability
IMT: intima-media thickness
MDRD: Modification of Diet in Renal Disease study
NCS: nerve conduction studies
PNP: polyneuropathy
QTc: corrected QT interval
SBP: systolic blood pressure
SD: standard deviations
T1DM: type 1 diabetes mellitus
T2DM: type 2 diabetes mellitus
UKPDS: The United Kingdom Prospective Diabetes
